# Nano-Magnetic Immunosensor Based on *Staphylococcus* Protein A and the Amplification Effect of HRP-Conjugated Phage Antibody

**DOI:** 10.3390/s150203896

**Published:** 2015-02-09

**Authors:** Xihui Mu, Zhaoyang Tong, Qibin Huang, Bing Liu, Zhiwei Liu, Lanqun Hao, Jinping Zhang, Chuan Gao, Fenwei Wang

**Affiliations:** Research Institute of Chemical Defence, State Key Laboratory of NBC Protection for Civilian, Beijing 102205, China; E-Mails: mxh0511@sohu.com (X.M.); qibin_huang@126.com (Q.H.); lbfhyjy@sohu.com (B.L.); liuzhw07@lzu.edu.cn (Z.L.); hlq70@163.com (L.H.); zjp337@126.com (J.Z.); g.ch.chuan@263.net (C.G.); wfwei2012@163.com (F.W.)

**Keywords:** *Staphylococcus* protein A, HRP-conjugated phage antibody, signal amplification, nanomagnetic immunosensor, *Staphylococcus aureus* enterotoxin B

## Abstract

In this research, super-paramagnetic Fe_3_O_4_ nanoparticles (magnetic particles) were coated with *Staphylococcus* protein A (SPA) and coupled with polyclonal antibody (pcAb) to construct magnetic capturing probes, and HRP-conjugated phage antibody was then used as specific detecting probe to design a labeled immunosensor for trace detection of *Staphylococcus aureus* enterotoxin B (SEB). The linear detection range of the sensor was 0.008∼125 μg/L, the regression equation was Y = 0.487X + 1.2 (*R* = 0.996, *N* = 15, *p* < 0.0001), the limit of detection (LOD) was 0.008 μg/L, and the limit of quantification (LOQ) was 0.008 μg/L. HRP-conjugated phage antibody, SPA and magnetic particles can enhance the sensitivity 4-fold, 3-fold and 2.6-fold higher, respectively. Compared with conventional double-antibody sandwich ELISA, the detection sensitivity of the sensor was 31-fold higher resulting from the integrated amplifying effect. The immunosensor integrates the unique advantages of SPA-oriented antibody as magnetic capturing probe, HRP-conjugated phage antibody as detecting probe, magnetic separation immunoassay technique, and several other advanced techniques, so it achieves high sensitivity, specificity and interference-resistance. It is proven to be well suited for analysis of trace SEB in various environmental samples with high recovery rate and reproducibility.

## 1. Introduction

In addition to the surface effect and volume effect of ordinary nanomaterials, magnetic nanoparticles (MNPs) also have super-paramagnetic effect, that is, even in a weak magnetic field they still show strong magnetism and disappear immediately after the withdrawal of the magnetic field, but now it is impossible for them to be permanently magnetized. Due to these magnetic properties, MNPs have been widely used in *in vitro* diagnosis, biological detection, biochips and biosensors, to simplify the operation procedures and improve detecting sensitivity, specificity and interference-resistance [1–5]. *Staphylococcus* protein A (SPA) can be linked with the Fc fragment of IgG molecules, whose Fab fragment are exposed outside, by hydrophobic interactions. Being better organized than direct physical adsorption or covalent binding, this oriented fixation has less impact on the activity of antibodies, and has now been widely used in the field of bio-detection [6–9]. The most widely used magnetic nanoparticles are iron oxides, such as γ-Fe_2_O_3_, and Fe_3_O_4_ whose critical size of superparamagnetism in magnetic fluid are 30 nm. By coupling SPA-coated super-paramagnetic Fe_3_O_4_ nanoparticles with polyclonal antibody (pcAb) in an oriented manner, not only the surface effect and super-paramagnetic nature of magnetic particles can be got, but also the capturing capacity, activity and separation efficiency of the probe can be improved, thus the target signal can be specifically amplified. In addition, the phage antibody shows both antigen-binding property and phage-like structure. A phage antibody contains multiple copies of capsid proteins (about 2700 copies of pVIII molecules), and can be bound with multiple anti-pVIII HRP-conjugated antibody molecules to form large phage antibody complex that contains a large number of HRPs, thus the signal of target molecule can be amplified greatly [10–14].

However, currently there is little understanding of the integrated amplifying effect of SPA-oriented fixed antibody, HRP-conjugated phage antibody, and super-paramagnetic nanoparticles. There is currently no report about the establishment of a rapid, sensitive, specific and interference-resistant immunosensor for SEB. In this study, the authors chose SEB as the target molecule, SPA-coated super-paramagnetic Fe_3_O_4_nanoparticles coupled with anti-SEB pcAb as the magnetic capturing probe, and HRP conjugated anti-SEB phage antibody as the specific signal detecting probe, to form a “magnetic capturing probe—SEB—HRP-conjugated phage antibody detecting probe” detecting scheme and establish a new way of nanomagnetic immunosensor for SEB detection. After comparison with other means of immunoassay, we focused on the amplifying effect of SPA-oriented antibody as the magnetic capturing probe and HRP-conjugated phage antibody as detecting probe. Unless bioterrorism and food poisoning incidents happened, it is difficult to get the actual source or real samples that were polluted by SEB. To provide technical support and reference for clinical diagnosis, environmental monitoring, and food hygiene inspection and anti-bioterrorism, this study focuses on the detection capability of the simulated sample, for example the river water, fertilized soil (organic matter content > 5%), butter biscuit (fat content > 30%) and whole rabbit blood as a matrix, which were polluted by SEB in our lab.

## 2. Materials and Methods

### 2.1. Reagents and Instruments

SEB standard substance, 1-(3-dimethylaminopropyl)-3-ethylcarbodiimide (EDC), *N*-hydroxysuccinimide (NHS), 2-*N*-morpholinoethanesulfonic acid (MES), SPA, bovine serum albumin (BSA), 3,3′,5,5′-tetramethylbenzidine (TMB), FeCl_3_·6H_2_O, FeCl_2_·4H_2_O, polyethylene glycol 6000 (PEG6000), 3-aminopropyltrimethoxysilane (APTES), and glutaric anhydride were purchased from Sigma (St. Louis, MO, USA). Abrin, ricin, carboxyl-modified super-paramagnetic Fe_3_O_4_ nanoparticles (15 nm), anti-SEB pcAb and anti-SEB phage antibody (M13KO7 phage display) were prepared in our lab. Horseradish peroxidase conjugated goat anti-mouse IgG (HRP-goat anti-mouse IgG) was purchased from Beijng Biosynthesis Biotechnology Co., Ltd. (Bejing, China). Horseradish peroxidase conjugated anti-M13 monoclonal antibody (HRP-anti-M13 mcAb) was purchased from GE Healthcare Europe GmbH (Freiburg, Germany). River water was obtained from Kunyu River (Bejing, China). Fertilized soil (organic matter content > 5%) was from Fenghuang Ridge (Bejing, China), Butter biscuit (fat content > 30%) was obtained from Master Kong, Ltd. (Bejing, China). Whole rabbit blood was obtained from venous blood of rabbit. The above four samples were all randomly collected from the environment.

*A_280nm_* and *A_450 nm_* values were respectively determined on BioMATE 3S UV-Vis spectrophotometer (Thermo Fisher Scientific Inc., Waltham, MA, USA) and Type 680 microplate reader (Bio-Rad, Hercules, CA, USA). Magnetic separation operation was carried out on 96-hole magnetic separation rack (Dynal Biotech GmbH, Hamburg, Germany).

### 2.2. Preparation of Carboxyl-Coated super-Paramagnetic Fe_3_O_4_ Nanoparticles

The authors adopted the microwave co-precipitation method to prepare super-paramagnetic Fe_3_O_4_ nanoparticles [15]. One gram of PEG6000 was dissolved by 150 mL ultrapure water in a triangular beaker, then 0.597 g of FeCl_2_·4H_2_O and 1.217 g of FeCl_3_·6H_2_O were added in, thoroughly mixed and then heated with a microwave oven for 30 s at a low heating power. Then some 1 M NaOH solution was added drop by drop to adjust the pH of the system to 11; the solution was heated in a microwave oven for another 30 s at low heating power, and then incubated in 80 °C water bath for crystallization for 30 min, washed with ultrapure water to neutrality and diluted to 50 mL. Anhydrous ethanol (50 mL) and APTES (1 mL) were added the solution and allowed to reaction at 60 °C for 10 h under the protection of nitrogen and with a stirring speed of 500 rpm/min. The product was then washed with anhydrous ethanol and double distilled water till no more oily suspension is produced, and then diluted with DMF to 50 mL. Glutaric anhydride (0.5 g) was added and allowed to react at 40 °C for 2 h with a stirring speed of 300 rpm/min. The final product was then washed with ultrapure water to neutral and diluted to 100 mL for later use. Meanwhile, the yield of solid product was calculated.

### 2.3. Preparation of SEB Magnetic Capturing Probe

Carboxyl-coated super-paramagnetic Fe_3_O_4_ (250 μL, 4 mg/mL) was washed with 0.01 M MES buffer (containing 0.5 M NaCl, 0.05% Tween-20, pH 4.7). Then 250 μL of 1 mg/mL EDC and 250 μL of 1 mg/mL NHS were added to allow to react at room temperature with stirring; followed by washing with 0.05 M borate buffer (BST, pH 8.5, containing 0.05% Tween-20 and 0.15 M NaCl) and magnetic-separation to discard the supernatant. A certain amount of SPA was added to allow to react at room temperature for 3 h with stirring, followed by washing with BST and magnetic-separation to discard the supernatant. BST (containing 10% BSA) was then added to block the remaining carboxyl groups on the magnetic particles and allowed to react at room temperature for 30 min with stirring, followed by washing with BST and magnetic-separation to discard the supernatant. A certain amount of anti-SEB pcAb was added to allow to react at room temperature for 2 h with stirring, the magnetic particles was then washed with BST, diluted to 1 mL and stored at 4 °C or −20 °C for long term storage.

### 2.4. Preparation of HRP-Conjugated Anti-SEB Phage Antibody Probes

Anti-SEB phage antibody (1 mL) with a titer of 1 × 10^11^ pfu/mL and 1 μL of anti-M13 HRP-conjugated mcAb were mixed, incubated at 37 °C for 1 h, and centrifuged at 9000 g for 20 min at 4 °C to remove the supernatant. The precipitate was then suspended with 0.01 M PBS buffer (containing 0.1%Tween-20, pH 7.4) and centrifuged to discard the supernatant. This process was repeated three times. Finally, the precipitate was resuspended in 0.01 M PBS buffer (containing 3% BSA, pH 7.4) and stored at 4 °C until use.

### 2.5. Establishment of the Nanomagnetic Immunosensor Based on SPA and HRP-Conjugated Phage Antibody

**Figure 1.**
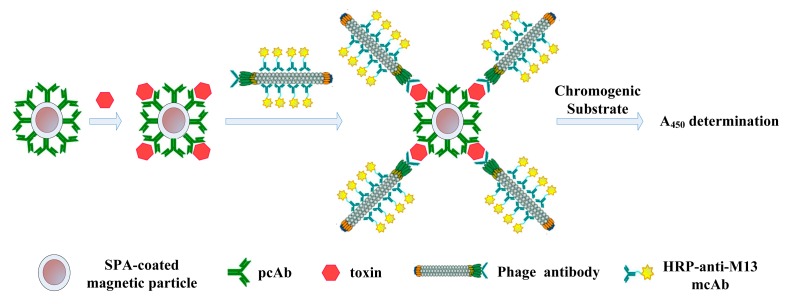
Model of toxin-detection by the nanomagnetic immunosensor based on SPA and HRP-conjugated phage antibody.

(1) Add samples: 100 μL of SEB magnetic capturing probe was added first. Then 100 μL of simulated sample or SEB standard substances of different concentrations, which were diluted with dilution buffer (0.01 M PBS buffer, pH 7.4), were added to each tube, except the blank and negative control tubes, into which 100 μL of carboxyl-coated superparamagnetic Fe_3_O_4_ nanoparticles or SEB magnetic capturing probe was added, respectively. Then the tubes were added with 100 μL of 0.01 M PBS buffer (containing 1% BSA, pH 7.4), allowed to react at 37 °C for 1 h, and then washed with PBST three times; (2) Add HRP-conjugated phage antibody probe: 100 μL of HRP-conjugated anti-SEB phage antibody was added to each tube except the blank tube, allowed to react at 37 °C for 1 h, then washed with PBST three times; (3) Add chromogenic substrate: 100 μL of chromogenic substrate was added to each tube, allowed to react at 37 °C for 15 min, then 50 μL of 2 M H_2_SO_4_ was added to terminate the reaction; (4) Record the absorbance value: supernatant was collected using the magnetic rack and detection at 450 nm. The whole process is illustrated in Figure 1.

### 2.6. Limit of Detection, Limit of Quantification, Linear Range and Specificity

Based on the calibration curve of the nanomagnetic immunosensor (Method 1), limit of detection, limit of quantification, linear range and other parameters of this method were determined, and 7.8 μg/L abrin, ricin, BSA and other non-target proteins were also analyzed using this method. In order to examine the specificity of this method, the results were compared with those of SEB.

In addition, under the same nanomagnetic immunosensor conditions, SPA-coated magnetic particle coupled with pcAb was respectively replaced by avidin-coated magnetic particles coupled with biotinylated pcAb and carboxyl-coated magnetic particles directly coupled with pcAb, to establish the standard curve of the nanomagnetic immunosensor based on HRP-conjugated phage antibody (Methods 2 and 3). Moreover, anti-SEB phage antibody was replaced by anti-SEB mcAb to establish the stand curve of the conventional double-antibody sandwich immunosensor (Method 4). The traditional double-antibody sandwich ELISA which applied pcAb-toxin- mcAb detecting pattern was also used to create the standard curve of double-antibody sandwich ELISA (Method 5). Through comparison of the nanomagnetic immunosensor method and conventional double-antibody sandwich ELISA, the signal amplification effects of SPA-oriented antibody, HRP-conjugated phage antibody and magnetic particles were investigated.

### 2.7. Measurement of Simulated SEB Samples

Fertilized soil (1 g, organic matter content > 5%), butter biscuit (1 g, fat content > 30%), whole rabbit blood (10 μL) and river water (1 mL) were added into 3.12 μL of 10 mg/L SEB standard. Then the mixture was diluted to 8 mL with dilution buffer (0.01 M PBS buffer, pH 7.4) to get a final SEB concentration of 3.9 μg/L. The river water samples were directly measured, and the fertilized soil samples were centrifuged at 5000 g for 20 min, while butter biscuit and whole rabbit blood samples were centrifuged at 10,000 g for 15 min and 10 min, respectively. The supernatants were then collected and the recovery rate, relative standard deviation and other indexes of the detection were analyzed and calculated.

## 3. Results

### 3.1. Preparation of Carboxyl-Coated Superparamagnetic Fe_3_O_4_ Nanoparticles

**Figure 2.**
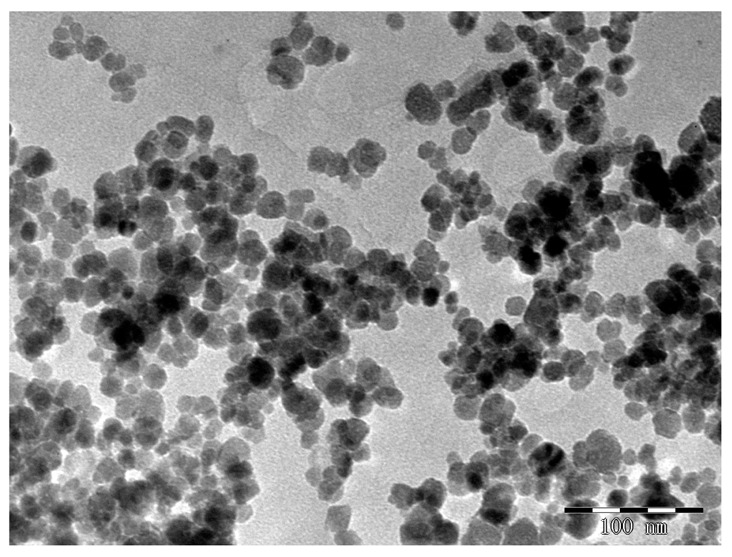
TEM image of carboxyl-coated super-paramagnetic Fe_3_O_4_ nanoparticles.

By combining microwave treatment with traditional chemical co-precipitation procedures, using microwave co-precipitation [15], our research group obtained carboxyl-coated superparamagnetic Fe_3_O_4_ nanoparticles with excellent dispersion, spherical morphology, particle size of 15 nm, and saturation magnetization of 78.875 emu/g. Compared with traditional co-precipitation methods, this method simplified the preparation of Fe_3_O_4_ magnetic nanoparticles and shortened the reaction time. Figure 2 shows a TEM image of carboxyl-coated super-paramagnetic Fe_3_O_4_ nanoparticles.

### 3.2. Preparation of Magnetic SEB-Capturing Probe

#### 3.2.1. Optimal Amount of Immobilized Anti-SEB pcAb

**Figure 3.**
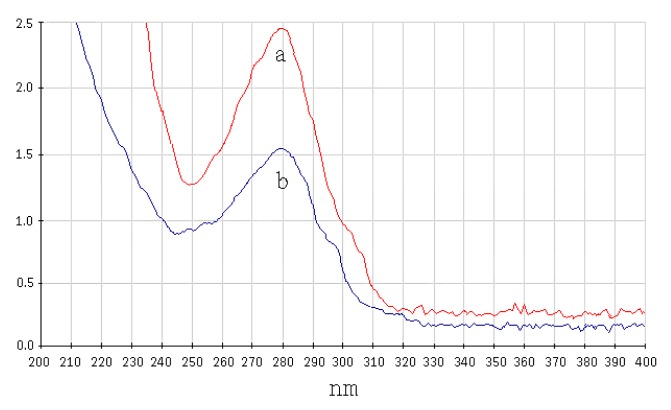
UV-Vis spectrum of SPA solution before and after binding to magnetic particles (a: UV-Vis spectrum of 400 μg of SPA solution before binding to magnetic particles; b: UV-Vis spectrum of 400 μg of SPA solution after binding to magnetic particles).

First, carboxyl-modified magnetic particles were activated by EDC-NHS to form NHS-active ester groupa, which spontaneously react with the amino group of proteins, so when a certain amount of SPA was added, it would immobilize on the surface of the magnetic particles. *A_280nm_* of the SPA solution was determined before and after immobilization, and the amount of immobilized SPA per mg of magnetic particles was calculated. As shown in Table 1, as the amount of added SPA was increased, the amount of immobilized SPA binding to magnetic particles gradually increased and tended to reach saturation. It was confirmed that the optimal amount of added SPA for 1 mg of magnetic particles was 400 μg, and the amount of immobilized SPA on 1 mg of magnetic particles was 151 μg. Figure 3 shows the UV-Vis spectrum of 400 μg SPA solution before and after binding to magnetic particles.

**Figure 4.**
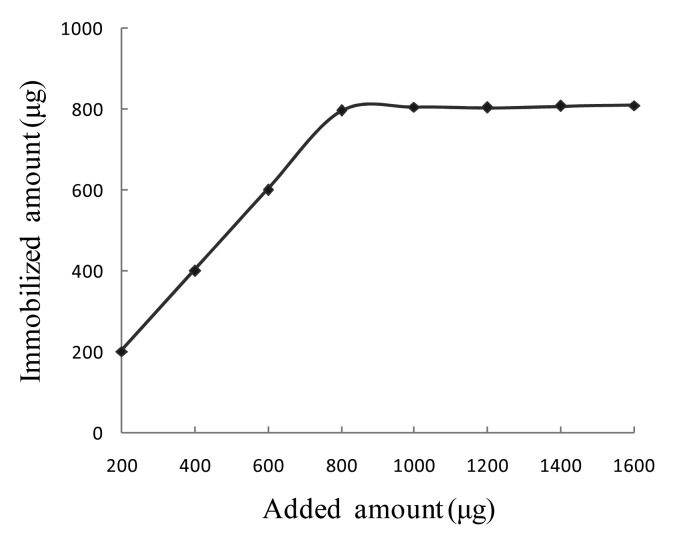
Immobilization ability of pcAb on SPA-coated magnetic particle.

Since SPA was specifically bound to the Fc fragment of IgG molecules, a certain amount of anti-SEB pcAb was added to get the magnetic SEB-capturing probe. *A_280nm_* of the anti-SEB pcAb solution was determined before and after immobilization, and according to the results, the actual immobilized amount of the anti-SEB pcAb was calculated to be 795 μg (Figure 4). Theoretically, the amount of immobilized anti-SEB pcAb to 1 mg of magnetic particle was 1115 μg as calculated according to the amount of immobilized SPA. The actual amount was 68.8% of the theoretical maximum. SPA is bivalent, which means that each SPA molecule can theoretically bind 2 IgG molecules. However, due to the steric hindrance effect, each SPA molecule bond only 1.4 molecules of anti-SEB antibody molecules in practice.

#### 3.2.2. The Magnetic Features of Magnetic SEB-Capturing Probe

**Figure 5.**
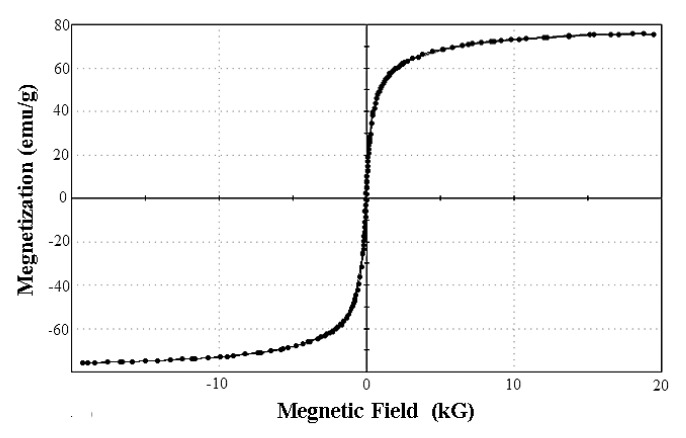
Magnetic hysteresis loops curve of the magnetic SEB-capturing probes.

The magnetic features of SEB-capturing probe was checked by VSM (see Figure 5), and the saturation magnetization of magnetic SEB-capturing probe was 75.687 emu/g. Compared with carboxyl-coated super-paramagnetic Fe_3_O_4_ nanoparticles, their saturation magnetization could be considered the same. The result showed that prepared SEB-capturing probes have good magnetic features.

### 3.3. Preparation of Enzyme Conjugated Anti-SEB Phage Antibody Probe

#### 3.3.1. Optimal Amount of HRP-Anti-M13 mcAb

**Figure 6.**
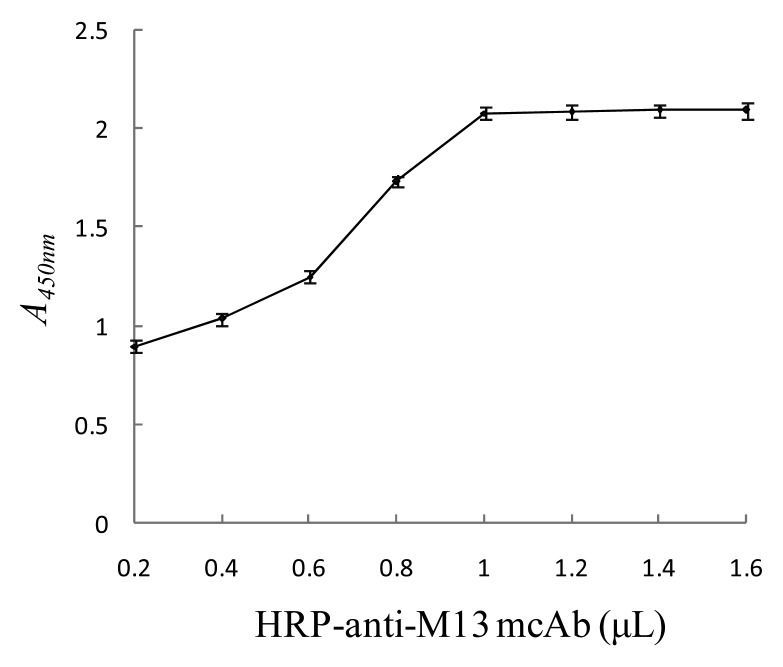
Determination optimum amount of HRP-anti-M13 mcAb.

To determine the optimal amount of HRP-anti-M13 mcAb, 0.2, 0.4, 0.6, 1.0, 1.2, 1.4 and 1.6 μL of HRP-anti-M13 mcAb was respectively mixed with 1 mL of anti-SEB phage antibody probe (1 × 10^13^ pfu/mL) to prepare HRP-anti-SEB phage antibody. The phage antibody (100 μL), SEB standard substance (100 μL, 125 μg/L), and magnetic SEB-capturing probe (100 μL) were mixed to react and form the nanomagnetic immunosensor. *A_450nm_* was tested for different amounts of HRP-anti-M13 mcAb to choose the optimal amount. As the amount of HRP-anti-M13 mcAb increased, *A_450nm_* gradually increased. When the amount of antibody reached 1 μL, the absorbance was steady, indicating that the HRP-anti-M13 mcAb bound to the anti-SEB phage antibody probe tended to be saturated, so the optimal amount of HRP-anti-M13 mcAb was 1 μL (see Figure 6).

#### 3.3.2. Determination of the Optimal Titer of the HRP-Conjugated Anti-SEB Phage Antibody

**Figure 7.**
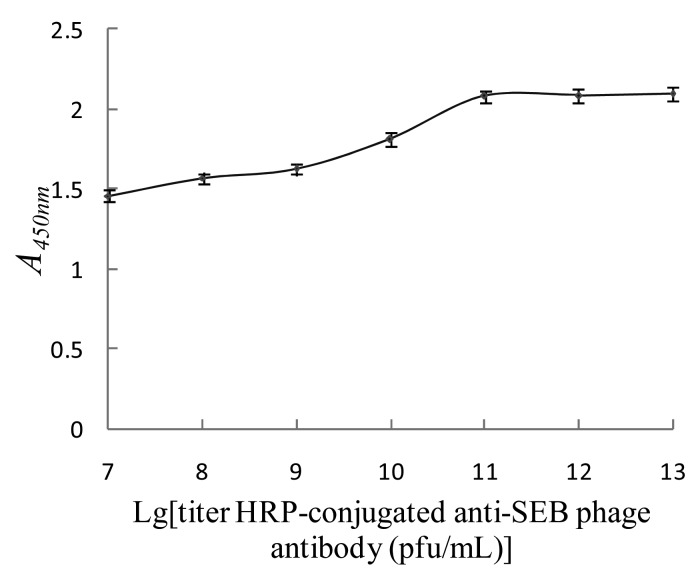
Determination optimum titer of HRP-conjugated anti-SEB phage antibody.

To determine the optimal titer of the HRP-conjugated anti-SEB phage antibody, 100 μL of magnetic SEB-capturing probe and 125 μg/L SEB standard substance were mixed and washed with PBST, then 100 μL of HRP-conjugated anti-SEB phage antibody with a titer of 1 × 10^7^, 1 × 10^8^, 1 × 10^9^, 1 × 10^10^, 1 × 10^11^, 1 × 10^12^ and 1 × 10^13^ pfu/mL were added. Then the nanomagnetic immunosensor was tested under the same conditions, and *A_450nm_* were determined for each test to determine the optimal titer of the HRP-conjugated anti-SEB phage antibody. As shown in Figure 7, as the titer of the HRP-conjugated anti-SEB phage antibody increased, *A_450nm_* gradually increased as well. *A_450nm_* reached a maximum and stopped rising when the titer was above 1 × 10^11^ pfu/mL, which was the optimal titer of the HRP-conjugated anti-SEB phage antibody.

#### 3.3.3. Determination of Activity of HRP-Conjugated Anti-SEB Phage Antibody Probe

**Figure 8.**
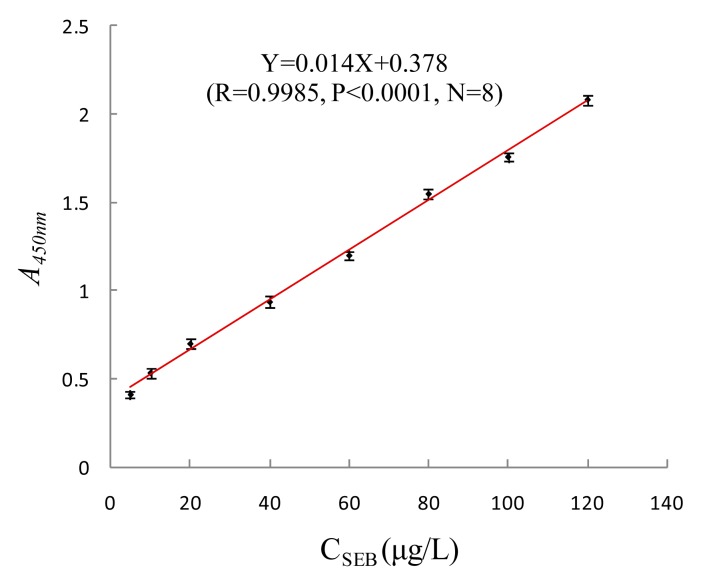
Determination activity of HRP-conjugated anti-SEB phage antibody.

To determine the activity of the HRP-conjugated anti-SEB phage antibody, microplates were coated with 5, 10, 20, 40, 60, 80, 100 and 120 μg/L SEB standard substance, and sealed with BSA, then 100 μL of HRP-conjugated anti-SEB phage antibody with a titer of 1 × 10^11^ pfu/mL were added. Under the same conditiona, indirect ELISA was used to determine the *A_450nm_*. According to the change of the *A_450nm_*, the activity of the HRP-conjugated anti-SEB phage antibody was checked. As shown in Figure 8, as the concentration of SEB increased, *A_450nm_* gradually increased as well, indicating that the probe can combine with the target toxin with a good dose-effect relationship. The concentration of SEB and *A_450nm_* showed a significant rectilinear correlation, and the regression equation was Y = 0.014X + 0.378 (*R* = 0.9985, *p* < 0.0001, *N* = 8). The results showed that prepared HRP-conjugated anti-SEB phage antibody with good activity can be used in the test.

### 3.4. Establishment of the Standard Curve for the Nanomagnetic Immunosensor to Detect SEB and the Detection Limit

**Figure 9.**
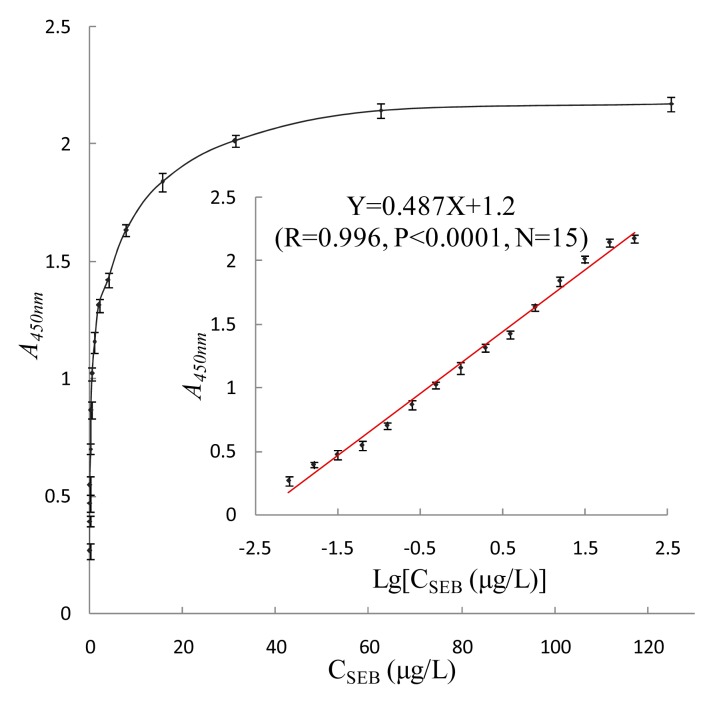
Standard curve of SEB determined by the nanomagnetic immunosensor.

The established nanomagnetic immunosensor was used to test 0.008∼250 μg/L SEB standard substance, and the results were plotted *vs*. SEB concentration on the X-axis and *A_450nm_* on the Y-axis (Figure 9). When the concentration of SEB was between 0.008 and 125 μg/L, the logarithm of SEB concentration and *A_450nm_* showed a significant rectilinear correlation, and the regression equation was Y = 0.487X + 1.2 (*R* = 0.996, *p* < 0.0001, *N* = 15).

The determination value, when higher than negative control *A_450nm_* value + 3SD and negative control *A_450nm_* value + 10SD, was deemed respectively as the limit of detection (LOD) and limit of quantification (LOQ). Average *A_450nm_* of twenty negative control samples was determined to be 0.124 ± 0.006, so the LOD was 0.008 μg/L, and the LOQ was 0.008 μg/L. In this research the standard deviation was tiny, showing this method has good accuracy and resulting in a LOQ close to the LOD.

**Figure 10.**
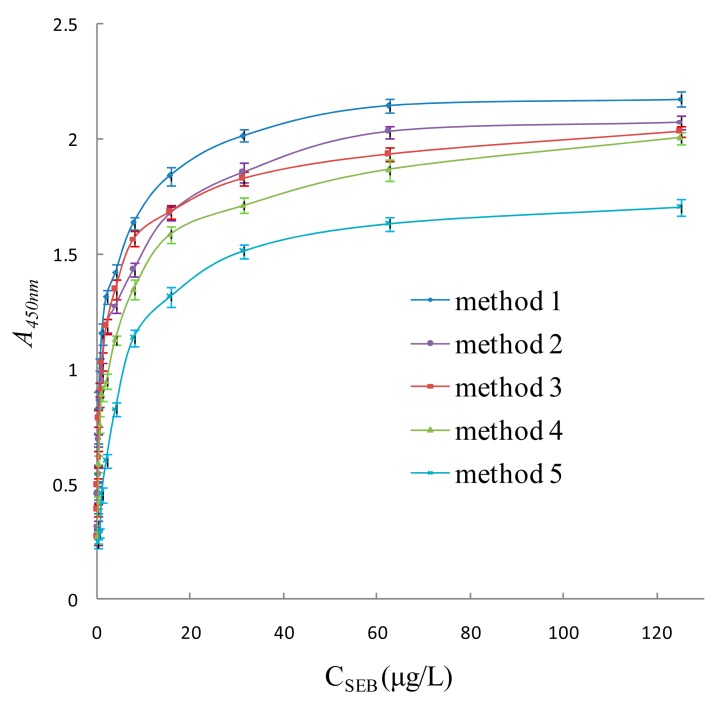
Comparison of four nanomagnetic immunosensors and ELISA (Method 1: SPA-coated magnetic particle coupled with pcAb capturing probe–toxins–HRP-conjugated phage antibody detecting probe detection scheme; Method 2: Avidin-coated magnetic particles coupled with biotinylated pcAb capturing probe–toxins–HRP-conjugated phage antibody detecting probe detection scheme; Method 3: Magnetic particle coupled with pcAb capturing probe–toxins–HRP-conjugated phage antibody detecting probe detection scheme; Method 4: Magnetic particle coupled with pcAb capturing probe–toxins–HRP-conjugated mcAb detecting probe detection scheme; Method 5: Conventional double-antibody sandwich ELISA).

Four nanomagnetic immunosensors and ELISA were compared, namely SPA-coated magnetic particle coupled with pcAb capturing probe–toxins–HRP-conjugated phage antibody detecting probe (Method 1), avidin-coated magnetic particles coupled with biotinylated pcAb capturing probe–toxins–HRP-conjugated phage antibody detecting probe (Method 2), pcAb-coupled magnetic particle capturing probe–toxins–HRP-conjugated phage antibody detecting probe (Method 3), pcAb-coupled magnetic particle capturing probe–toxins–HRP-conjugated mcAb detecting probe (Method 4) and conventional double-antibody sandwich ELISA (Method 5). As shown in Figure 10 and Table 2, HRP-conjugated phage antibody can enhance sensitivity 4-fold higher from comparison between Methods 3 and 4, SPA can enhance sensitivity 3-fold higher from comparison between Methods 1 and 3, magnetic particles can enhance sensitivity 2.6-fold higher from comparison between Methods 4 and 5. The nanomagnetic immunosensor based on SPA and HRP-conjugated phage antibody integrated the three amplifying effects above, and the detection sensitivity was 31-fold higher than a conventional double-antibody sandwich ELISA. It was also proved that SPA can orient and orderly arrange antibodies on magnetic particles, improving the efficiency and detection sensitivity of the magnetic capturing probe. The magnifying effect of HRP-conjugated phage antibody and magnetic particle in the nanomagnetic immunosensor amplified the target signal and improved the sensitivity of detection greatly.

### 3.5. Accuracy and Specificity

Within the linear concentration range, 62.5, 7.8, 0.98 and 0.016 μg/L of SEB standard substance was tested by using nanomagnetic immunosensor. Each concentration was tested five times to obtain the *A_450nm_* values, which were 2.144 ± 0.029, 1.634 ± 0.024, 1.156 ± 0.046 and 0.394 ± 0.021 with RSDs of 1.33%, 1.47%, 4.0% and 5.34%, respectively, showing good accuracy.

This nanomagnetic immunosensor was also tested with 7.8 μg/L SEB, abrin, ricin and BSA (Table 3) whose matrix was dilution buffer (0.01 M PBS buffer, pH 7.4), and the negative control was the simulated sample without SEB, for example, dilution buffer, river water, fertilized soil (organic matter content > 5%), butter biscuit (fat content > 30%) and whole rabbit blood, which separately acted as matrix. The *A_450nm_* values of the non-target proteins, abrin, ricin, and BSA were close to negative results, indicating high specificity of this method to detect SEB.

### 3.6. Detection of SEB in Simulated Samples

As shown in Table 4, all simulated samples which contained SEB in river water, fertilized soil (organic matter content > 5%), butter biscuit (fat content > 30%) and whole rabbit blood were examined, showing recovery rates above 90%. This method met the requirements for analysis of the simulated samples above, showing high recovery rates and reproducibility.

## 4. Discussion

As a new functional material, magnetic nanoparticles have shown good prospects in biomedicine, molecular biology, immunology, cell biology and environmental engineering. Magnetic nanoparticles are superparamagnetic when their diameter was less than 30 nm, while the carboxyl-coated Fe_3_O_4_ nanoparticles had a diameter of 15 nm and were superparamagnetic under an external magnetic field, so they were both superparamagnetic nanoparticles. Compared with magnetic microparticles, the carboxyl-coated Fe_3_O_4_ nanoparticles show more significant surface area and volume effects. The surface area of the particle was dramatically increased; the density of functional chemical groups, selective absorption capacity, chemical reactivity, and stability were enhanced; and the time to reach absorption equilibrium was shortened. Due to these features, carboxyl-coated superparamagnetic Fe_3_O_4_ nanoparticles coupled with pcAb as the vector of the magnetic capturing probe would be superior to microparticles.

This study used SPA-coated magnetic particles coupled with pcAb as magnetic capturing probe, HRP-conjugated phage antibody as detection probe, to establish a nanomagnetic immunosensor to detect SEB. A phage antibody has multiple copies of capsid proteins which can be bound by the corresponding HRP-conjugated antibody. Therefore, compared with conventional primary antibody—HRP-conjugated secondary antibody scheme, such a HRP-conjugated phage antibody can be conjugated with more HRP molecules and generates an amplifying effect when being used as a detecting probe. In a previous study, our research group has established the avidin-coated magnetic particles coupled with biotinylated pcAb as capturing probe—toxins—HRP-conjugated phage antibody as detecting probe detection scheme (Method 2) [16], which was also a sandwich magnetic immunosensor and had a LOD of 0.016 μg/L. Based on these circumstances, in this study, we further incorporated SPA that fixes antibodies in an oriented manner by using SPA-coated magnetic particles coupled with pcAb as capturing probe, and use HRP-conjugated phage antibody as detection probe, to establish this method of SPA and HRP-conjugated phage antibody-based magnetic immunosensor, which showed 2-fold higher detection sensitivity (the LOD reached 0.008 μg/L.) by improving the efficiency of the probe.

Both methods were carried out under the same magnetic separation immunosensor conditions. Each milligram of avidin-coated magnetic particles was coupled with 987 μg of anti-SEB pcAb in practice, that is, 40.2% of the theoretical maximum of 2300 μg. Theoretically, an avidin molecule can bind four biotin molecules, however, due to steric hindrance, an avidin molecule bound only 1.7 molecules of biotinylated anti-SEB pcAb. Moreover, each milligram of SPA-coated magnetic particle fixed 785 μg of anti-SEB pcAb, which meant that a SPA molecule bound 1.4 molecules of anti-SEB pcAb. Even though SPA-coated magnetic particles fixed less pcAb than avidin-coated particles, it achieved higher probe efficiency and higher detection sensitivity, because SPA couples antibody molecules in an oriented manner.

## 5. Conclusions

This paper proposed a new labeled immunosensor for biological trace sample detection. The immunosensor integrates the unique advantages of an SPA-oriented antibody as magnetic capturing probe, HRP-conjugated phage antibody as detection probe, magnetic separation immunoassay technique, and several other advanced techniques. Such integration idea shows good application prospects for the detection of complex biological samples that require high sensitivity, specificity and interference-resistance. It is proven to be well suited for analysis with high recovery rates and reproducibility of trace SEB in various environmental samples. The linear range of the sensor was 0.008∼125 μg/L, the LOD was 0.008 μg/L, and the LOQ was 0.008 μg/L. HRP-conjugated phage antibody, SPA and magnetic particles can enhance sensitivity by 4-fold, 3-fold and 2.6-fold, respectively. Compared with a conventional double-antibody sandwich ELISA assay, the detection sensitivity of the sensor was 31-fold higher as a result of the integrated amplifying effect.

## Figures and Tables

**Table 1.** Absorbance value at 280 nm of SPA solution before and after binding to magnetic particle.

**Added Amount (μg)**	***A****_280nm_* **before**	***A****_280nm_* **after**	**Binding Rate (%)**	**Immobilized Amount (μg)**
40	0.428 ± 0.005	0.078 ± 0.003	81.8	33
80	0.773 ± 0.006	0.152 ± 0.004	80.3	64
160	1.077 ± 0.007	0.516 ± 0.006	52.1	83
240	1.498 ± 0.005	0.818 ± 0.005	45.4	109
320	1.959 ± 0.008	1.189 ± 0.007	39.3	126
400	2.443 ± 0.006	1.520 ± 0.006	37.8	151
500	3.037 ± 0.009	2.137 ± 0.008	30.7	154
550	3.446 ± 0.007	2.486 ± 0.007	27.9	153

**Table 2.** Comparison of four nanomagnetic immunosensor and ELISA (Methods 1∼5 see Figure 8).

**Detecting Scheme**	**Linear Range (μg/L)**	**Regression Equation**	**Correlation Coefficient (R)**	**Limit of Detection (μg/L)**	**Detection Time (h)**
Method 1	0.008∼125	Y = 0.487X + 1.2	0.9960	0.008	2.5
Method 2	0.016∼125	Y = 0.497X + 1.048	0.9955	0.016	2.5
Method 3	0.024∼125	Y = 0.48X + 1.068	0.9980	0.024	2.5
Method 4	0.096∼125	Y = 0.542X + 0.878	0.9970	0.096	4
Method 5	0.25∼250	Y = 0.583X + 0.525	0.9894	0.25	4

**Table 3.** Detection specificity of the nanomagnetic immunosensor (*n* = 5).

**Object**	***A****_450nm_*	**Relative Standard Deviation (%)**
SEB	1.636 ± 0.026	1.62
Abrin	0.123 ± 0.008	6.68
Ricin	0.125 ± 0.006	4.63
BSA	0.122 ± 0.008	6.18
River water	0.123 ± 0.005	4.19
Fertilized soil	0.125 ± 0.004	3.58
Butter biscuit	0.124 ± 0.006	5.26
Whole rabbit blood	0.128 ± 0.006	4.94
PBS buffer	0.120 ± 0.006	4.97

**Table 4.** Determination of the simulated SEB specimens (*n* = 4).

**Sample**	**Added (μg/L)**	**Found (μg/L)**	**Recovery (%)**	**Relative Standard Deviation (%)**
River water	3.9	3.66 ± 0.12	93.9	3.25
Fertilized soil	3.9	3.59 ± 0.14	92.1	3.89
Butter biscuit	3.9	3.55 ± 0.09	90.9	2.63
Whole rabbit blood	3.9	3.52 ± 0.08	90.2	2.34
